# Chicago Veterinary College

**Published:** 1896-05

**Authors:** 


### COMMENCEMENT EXERCISES.

### OHIO VETERINARY COLLEGE.

### CHICAGO VETERINARY COLLEGE.

On March 24th the Thirteenth Annual Commencement Exercises were held in the College auditorium. On the evening previous a banquet was tendered by the trustees to the faculty and graduating class at the Sherman House, and a most enjoyable evening was spent. The week preceding the date of the graduating exercises was taken up by the final examinations, the results of which were that twenty-eight students passed. Of this number, eight passed in honors. Dr. E. J. Davidson received the gold medal of the college for the highest general average. Dr. Davidson also received the prize for the highest standing in theory and practice. Dr. E. Jentzsch won the prize for the best standing in anatomy, and Dr. Walter Bolton the prize for the best examination in chemistry.

On the evening of the commencement exercises a large audience assembled in the auditorium of the college building. The lecture-room was tastefully decorated for the occasion, and many beautiful floral tributes were brought by admirers of the successful students. The chair was occupied by Dr. A. H. Baker. The other members of the faculty occupying the rostrum were Jos. Hughes, M.R.C.V.S.; Finley Ellingwood, M.D.; C. E. Sayre, M.D., D.V.S.; J. F. Ryan, M.D.C.; A. R. Edwards, M.D.; Geo. S. Baker, D.V.S.; E. L. Quitman, M.D.C.; M R. Trumbower, D.V.S.; and A. S. Alexander, V.S.F.H.A.S. Among the visiting veterinarians who occupied seats on the platform were Drs. Olof Schwarzkopf, C. A. White, A. McBride, F. S. Lambert, A. Savage, N. G. Bailey, and others. The Chairman commenced the proceedings by reviewing at length the progress of the institution since its organization in 1883, and in the course of his address stated that this was a most fitting time to announce that henceforth the college course would be extended from two to three sessions, covering three years instead of two as heretofore. He wished at the same time to make the statement that the Chicago Veterinary College eight years ago was the first of the two-year schools to agitate the question of extending the course to three years. At that time this school corresponded on this subject with some of the most prominent of the older two-year schools, but our communications were with one exception entirely ignored, and even in the case of the exception the proposition was declared premature.

Having congratulated the members of the graduating class on their successful completion of the course, he conferred on each member the degree of Doctor of Comparative Medicine and presented the diplomas. The class valedictorian, Dr. A. C. Worms, then delivered his address, and was followed by Dr. A. S. Alexander on behalf of the faculty. Dr. E. P. Flack delivered the class prophecy. Dr. Schwarzkopf then addressed the faculty and graduates, and in doing so he highly complimented them on the change from a two to a three-session course. He predicted that it would materially add to the prestige which the Chicago Veterinary College already had.

The following is the list of graduates and their present location: W. Bolton, Waukesha, Wis.; R. L. Brown, Janesville, Wis.; C. E. Brownback, Pleasant Plains, Ill.; O. F. Butterfield, Leithton, Ill.; E. J. Davidson, Grand Forks, N. D.; Edward Dickinson, Fayette, Iowa; G. K. Dodson, Los Angeles, Cal.; F. A. Dolton, Dolton, Ill.; Henry F. Eckert, Reeseville, Wis.; E. R. Flack, Milwaukee, Wis.; J. W. Foster, La Fayette, Ind.; D. J. Halloran, Hortonville, Wis.; E. M. Herrin, Highland, Ill.; J. G. Hope, Chicago, Ill.; E. Jentzsch, Chicago, Ill.; E. A. Johnson, Platteville, Wis.; M. H. Kyle, Highland, Ill.; N. W. Kyle, Colfax, Ill.; J. A. McGarry, Milwaukee, Wis.; W. J. Malone, Springdale, Wis.; F. McCoy, Lake Odessa, Mich.; C. G. Nelson, Chicago, Ill.; J. H. Pear, Chicago, Ill.; F. R. Pierce, Dows, Iowa; F. P. Scott, Petersburg, Ill.; Wm. Voss Kiel, Wis.; C. H. Whitwell, Dubuque, Iowa; A. C. Worms, Chicago, Ill.

